# Basic business knowledge scale for secondary education students. Development and validation with Spanish teenagers

**DOI:** 10.1371/journal.pone.0235681

**Published:** 2020-07-07

**Authors:** Antonio Bernal-Guerrero, Antonio Ramón Cárdenas-Gutiérrez, Elisabet Montoro-Fernández

**Affiliations:** Department of Theory and History of Education and Social Pedagogy/Faculty of Education Sciences, Seville University, Seville, Spain; Aalborg University, DENMARK

## Abstract

**Introduction:**

In the current international context, entrepreneurship education claims a privileged place within educational systems, given that it contributes decisively to innovation and to the set of competences demanded in the new knowledge-based economy. The state of the research in this line highlights the existing formative deficiencies at these basic education levels, despite the fact that numerous initiatives of fostering business culture have already been developed. Among the currently existing gaps, conspicuous by its absence is the lack of instruments capable of efficiently measure the basic business knowledge, needed to progressively constitute a consistent business identity stands out. In this sense, we set ourselves the construction and validation of a basic business knowledge scale for the Secondary Education stage.

**Methods:**

This study was implemented in two phases. In the first phase, the dimensions and components of the Basic Business Knowledge Scale were identified via a systematic review of the literature following the PRISMA-P protocol and a qualitative study. In the second phase, the scale was developed and validated. On the one hand, a content validation was conducted through interviews of experts and students, studying the content validity (the Content Validity Ratio and the Content Validity Index) and the face validity (Think-aloud protocols). On the other hand, the construct validity was analyzed through an Explanatory Factor Analysis (EFA) and a Confirmatory Factor Analysis (CFA). Then, the reliability was calculated with the Cronbach Alpha and the test stability with a test-retest. The convergent validity has been validated by the average variance extracted (AVE) and the discriminant validity between constructs was established through the AVE estimated for each construct with the squared interconstruct correlations associated with that factor. The sample was made up of 1440 students (679 girls and 761 boys) from age 11 to 17 (M = 14.6, SD = 1.597).

**Results:**

The EFA and the CFA showed evidence of a first-order three-factor structure (Knowledge in Business Management (KBM), Legal Knowledge (LK) and Strategic Knowledge (SK)), and a second-order factor, Basic Business Knowledge. In the construct validity two items were eliminated due to their factor loadings being lower than .40. The results of the fit indices contributed acceptable values regarding the proposed model. The three subscales and the scale as a whole revealed a satisfactory internal consistency with Cronbach alphas over .75. The intraclass correlation coefficient (ICC) was above .90, showing an appropriate stability. The convergent validity offers values over .80 in the composite reliability (CR) and the average variance extracted (AVE) is greater than .50. Moreover, in the divergent validity, the values of the square root of the AVE are greater than the correlations with the other constructs. Finally, the Basic Business Knowledge Scale has 18 items.

**Conclusions:**

We find evidence concerning the validity and reliability of the Basic Business Knowledge Scale, tested with Spanish Secondary Education students within the compulsory stage of teaching. We believe that this Scale can contribute to a better understanding of the formation of indispensable basic culture to establish a genuine business spirit.

## Introduction

The formation of entrepreneurial competence is becoming a requirement within the current conditions of development, characterized by an emphasis on innovation, a high level of interactive skills and the priority of knowledge [1]. Universities have the responsibility of playing a key role in the formation of qualified specialists with high creative and intellectual potentials and capable of implementing viable business projects, therefore becoming drivers of a sustainable economic growth. That way, the great existing interest in the current universities concerning the business culture development practices and the entrepreneurial literacy of their students is thus understood [2] [3]. Yet, prior to that and essential for the configuration of a business identity, it is necessary the establishment within compulsory education, with permanent vocation, of a knowledge system in the field of entrepreneurship [4].

It is necessary to progressively and effectively cultivate business education from primary school, especially within the framework of a postmodern society where people have to assume the role of their lives like never before [5], as they do not encounter closed living programs as took place in traditional societies. In this sense, non-cognitive factors [6] [7], such as motivation and leadership, creativity and innovation, proactivity and autonomy, become essential for the formation of business identity and economic grouth of society, togheter with properly entrepreneurial knowledge and behavior [8]. Although there are differences concerning the pedagogical focuses applied and about what indicators more clearly reflect success in formation, there appears to be a consensus regarding the need to boost a unique framework of entrepreneurship education [9] as a new and fruitful way for the promotion of economic development.

Indeed, business education can be strategically promoted and strengthened in multiple ways [10]. Amongst others recently formulated, with practices of coproduction, involving from an early age business environments in the formal educational process [11], or raising a direct contact with the business environment through the methodology of projects [12]. This also occurs through the use of certain advanced technological tools [13] [14], directly cultivating self-efficacy as a predictor of business behavior [15], using autonomous learning in a business context, trying to drive employability [16], underlining the relevance of finding business opportunities [17] [18] [19] and linking them with sustainable development, permeating them with moral strength, beyond the appropriation of tangible business results [20] [21]. Likewise, this takes place indirectly cultivating a person’s entrepreneurial qualities, without associating them in the first instance with the creation of enterprises and businesses [22], as well as relating certain entrepreneurial skills with the design and development of life projects [23].

Although a model of business competence established for a specific educational system is difficult to adjust to the needs and objectives of other educational systems [24], we consider that there is a certain potential of transfer between the diverse models compared. Entrepreneurial competence requires specific attitudes, skills and knowledge, although a diversity of possible developments can be admitted according to the diverse business and economic contexts which we refer to. In a recent study [25], combining the established theory of planned behavior [26] and the model of “iceberg competences” [27], five established areas determine the appearance of entrepreneurial intention: motives, features, self-concept, skills and knowledge. The need for knowledge is also claimed, as a “framework of qualifications” and implicitly so in other domains which are configurators of competence [28].

In spite of having minimized the value of cultural contents from functionalist approaches, in many predominant cases learning without disciplinary knowledge is fading. Not all knowledge is dispensable or substitutable by any other, given that it is not a question of solely developing certain aptitudes or mental capacities, attitudes or skills, geared to the generation of innovation and initiative [29], but rather confirming a solid cultural formation around the subject upon which to build a specific business identity [30]. Ken Robinson [31], one of the most influential current authors about the essential educational value of creativity, proposes, to overcome the outdated educational system inherited from the Industrial Revolution, a personalized approach of education with the aim of harnessing the potential of available technological and professional resources and of fostering the participation of students for them to lose fear of making mistakes and develop their passion for learning and for establishing their creativity. Nevertheless, he considers that none of this is possible without a balanced vindicating of the value of disciplinary knowledge.

Indeed, the lack of formation in contents linked with business management, with legal aspects associated to business organizations and with the formalization of businesses, as well as with certain basic and strategic aspects for the creation of enterprises and their consolidation, can hamper the entrepreneurial initiatives taken outside the educational system. And this set of knowledge is indispensable, even though we use alternative strategies to achieve it [32].

Some recent studies reveal that secondary schools students are insufficiently able from a practical point of view to survive in the real economic world and to undertake their own business activities [33]. For example, the limited attention paid to financial education in formal education weakens the entrepreneurial capacity of adult people. This eventuality has been extensively analyzed in the literature, showing the incidence of financial education in entrepreneurship, which is remarked in a broad bibliometric study that spans the last three decades about the global trends of research concerning the effect of financial formation on the creativity of individual entrepreneurship [34].

The current situation calls for the substantive and functional incorporation in entrepreneurship education, within primary and secondary schools, of fundamental specific contents for the configuration of an entrepreneurial identity linked to ethical principles for a sustainable economic development of society [35] [36], as an effective way of forming future entrepreneurs. The assertion of specific knowledge linked with entrepreneurship does not necessarily mean the choice of a traditional didactic methodology, since pedagogical innovation is not incompatible with the formative contents finally selected [37] [38].

We are witnessing a considerable amount of proposals of entrepreneurship education, of a diverse kind and scope, trying to drive business culture. The majority of countries have specific programs, projects and pedagogical experiences related with entrepreneurship, most of which have been developed in the higher levels of education and for adults in a diversity of extracurricular ways. Progressively, this attention has also been aimed, with profusion and intensity, not only at the university increasing level but also at the primary and secondary stages of education. For the most part, the integration of entrepreneurial competence in schools has been conducted through the creation and the functioning of microenterprises, or through the development of subjects specifically created to do so. It still remains a long way to go in the formation of entrepreneurship in schools, therefore being necessary the deepening, together with certain attitudes and skills contained in the proactive management of projects and their consequences, on the knowledge of the functioning of the economy and recognizing the opportunities and challenges that entrepreneurs must face in the real world [39].

However, the set of initiatives, actions and programs for the promotion of business culture in schools suffers from a lack of rigorous evaluations of its real achievements and their quality [40]. Although there have been some relevant contributions, we are missing, in general, precise and sufficient diagnoses about its effectiveness. Particularly, there is a notable gap in the diagnosis of the precise knowledge for the configuration of a business profile, or rather proto-business in the compulsory teaching levels, this being a scarcely applied way of assuring the future success of business initiatives.

We believe the design of an assessment instrument about business knowledge to be a suitable response to this situation. This instrument would facilitate the research and understanding of business knowledge related with the shaping of a business identity. The development of a scale which measures business knowledge in adolescence is necessary to orientate, get to know and assure appropriate educational actions related with entrepreneurship education at this age. The aim of this study is specifically to formulate a valid and reliable basic business knowledge scale for the stage of Compulsory Secondary Education (Middle School and High School).

## Materials and methods

### Study design

**Fig 1:**
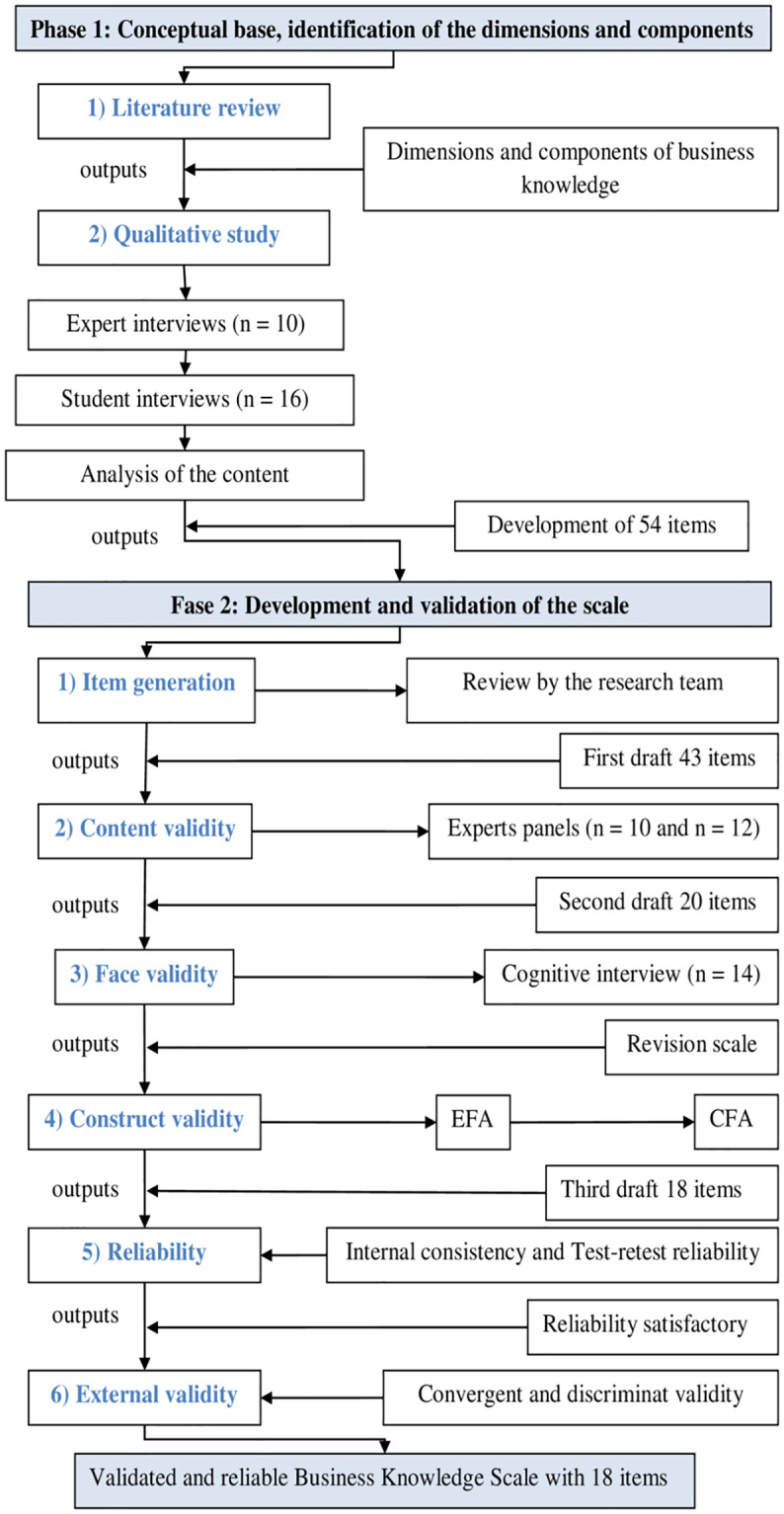
Summary of the process employed for the development and validation of the Basic Business Knowledge Scale (BBKS).

Following the Standards for Educational and Psychological Tests [41], this research has been carried out in two phases [42] [43] [44]: 1) the identification of the dimensions and components of business knowledge in the stage of Compulsory Secondary Education; 2) the development and validation of the scale of business knowledge in adolescence (Fig 1).

#### Phase 1: Conceptual base, identification of the dimensions and components

**Fig 2:**
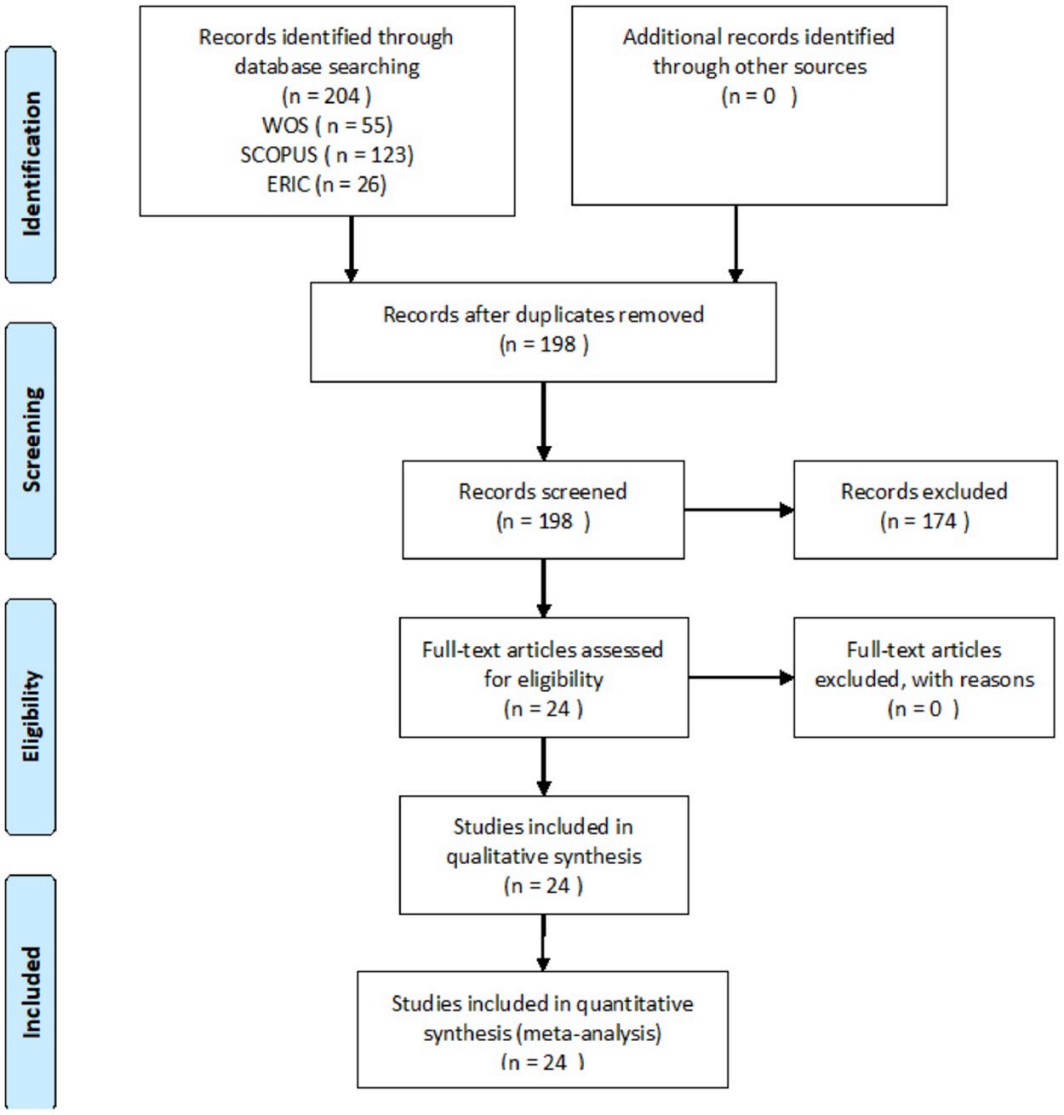
Flow diagram concerning the article selection process. Adapted from Moher et al. [46].

**1) Literature review**. The aim was to define the dimensions and components of introductory and fundamental business knowledge. To do so, a systematic bibliographic review was done along with a selection of studies following the PRISMA-P protocol [45] [46]. The PRISMA-P protocol comes from medical research and has recently begun to be applied in literature reviews in the field of education, to obtain greater rigor and methodological consistency [47]. However, its use in educational bibiometric research implies that not all of its parts are applicable, since clinical trials are not reviewed. Furthermore, in the case of this study, where the systematic review that has been carried out is not the purpose of the research, but a phase of it, since the ultimate purpose is the construction and validation of a questionnaire. Thus, four items corresponding to the Methods and Results sections of the PRISMA-P protocol are applied, because the remaining items are not applicable to this research (S1 File). The search, identification and selection were carried out in two phases. In the first phase, an expert author in literature reviews searched in the databases of Web of Science, ERIC and Scopus with the following equation: (“Entrepreneur* education programs” OR “business education programs”). The criteria of the bibliographic search were articles in scientific journals with publication dates from January 1st. 2000 to 31st. December 2017, being identified 204 potential articles. In the second phase, three independent researchers assessed the potential articles which had some of the following terms in the title and in the abstract: a) business knowledge; b) entrepreneurship education and entrepreneurial education or business education; c) compulsory secondary education; and d) Middle School and High School. We established two inclusion criteria which in the end guided the bibliographic selection carried out in the first phase: a) Type of articles: research works related with theoretical, descriptive, evaluative, qualitative and mixed studies were included; and b) type of population: research works centered on students in the formal education stages were taken into account. As a criterion for exclusion we adopted the one of studies related with research works in non-formal education. 6 duplicated studies were eliminated and 174 articles were excluded. Thus, 24 articles were selected for the thematic and inductive analysis of the content via a codification system [48] (Fig 2). The Kappa index of Fleiss was calculated to achieve a concordance between the researchers [49] obtaining a score of K = .82, considered as a very good value [50] [51].

**2) Qualitative study**. Via a qualitative study, 10 in depth interviews were done with experts in entrepreneurship education in the stages of compulsory education to find out their perceptions, knowledge and teaching experiences related with business knowledge taught in the programs of entrepreneurship education or business education (S2 and S3 Files). The selection criteria were to have developed these programs during a minimum of two academic years in Compulsory Secondary Education centers and to have been their coordinators. Also, 16 exhaustive interviews were conducted with secondary education students (S2 and S3 Files). The selection criterion was to have taken part in those programs during at least one school year. The aim of interviewing the students was to get to know what typology and which main characteristics the knowledge contained in the programs had. All the interviews were recorded, transcribed and codified via a system of categories to find out the components of the business knowledge and were done from 1st. February to 29th. March 2018. Each of them lasted from 30 to 45 minutes. An analysis of the content of the interviews was made using the NVivo 11 Plus for Windows software. The scripts were scrutinized by fifteen experts who analyzed their validity and the pertinence of the items. The experts, selected for their recognized academic and professional interests in the area of entrepreneurship education, came from diverse sectors: the university area (7), educational centers (5) and organizations or foundations of the civil society (3). To avoid the disaggregating effect of individual valuations [52] [53], a search was established for the maximum level of consensus between the experts themselves. To do so, it was agreed that a level of consensus above 80% had to be achieved and that there had to be an acceptable degree of homogeneity in the answers (coefficient of variation less than 80%) in the two rounds of participation. Later, the research team discussed and reflected about the content of the interviews, finishing the analysis when the saturation of the information was attained and new topics did not arise.

In this first phase an initial set of 54 items for 3 dimensions was generated, emanating from both the systematic review of the selected documents and the qualitative study carried out: Knowledge in Business Management (KBM), Legal Knowledge (LK) and Strategic Knowledge (SK).

#### Phase 2: Development and validation of the scale

**1) Item generation**. The 54 items generated in Phase 1 were carefully reviewed by the research team, and a total of 11 items were considered superfluous and therefore eliminated; 43 being kept. As to the type of answer, there is not a consensus concerning the categories of the Likert scale which must be used [54]. In our case, the scale has been designed for adolescents, taking into account the age of the studied group and, to avoid confusion while filling out the questionnaire, it was decided to adopt a three-value Likert scale: 2,“It has been worked on at class and I think I’ve learnt it” (Worked and learned, WL); 1, “I think it has been worked on at class, but I haven’t learnt it” (Worked but not learned, WNL); 0, “I think it hasn’t been worked on at class and I haven’t learnt it” (Not worked, NW).

**2) Content validity**. In a first stage, for the content validity, a panel of experts made up of twelve specialists in entrepreneurship education, business management, firm projects, marketing, corporate social responsibility and economics carried out a first review about formal questions, the drafting and the scaling of the test. In a second stage, the Content Validity Ratio (CVR) and the Content Validity Index (CVI) were calculated, asking the experts about the need (CVR), relevance, clarity and simplicity (CVI) of each of the items. Another twelve experts calculated the CVR, evaluating each item with a Likert scale with the following values: 1 = essential; 2 = useful, but not essential; 3 = not essential. Based on the Lawshe scores, the items with a score equal to or greater than .56 were kept in the scale [55] [56]; being eliminated 17 items in this process. The CVI [57] [58] of each item was calculated on a 4-point Likert scale (1 = not relevant; 2 = somewhat relevant; 3 = quite relevant; 4 = highly relevant [59], and all the items which had a value equal to or higher than .80 were kept. In the CVI, 6 items were eliminated for not fulfilling this criterion. Thus, 20 items remained to carry out the factor analysis. The total scores of the CVR and the CVI were .88 and .93, respectively, revealing a good degree of content validity [59].

**3) Face validity**. Cognitive interviews were used to establish the face validity of the scale [60] [61] [62]; specifically, the Think-aloud protocols [63] [64] [65] [66] [67]. 14 participants were selected by a convenience sampling, of similar characteristics to the study aim group, aged between 11 and 16 years old from the Compulsory Secondary Education (ESO) stage and with the following distribution of participants per course: 1° ESO (4 students), 2° ESO (4 students), 3° ESO (3 students) and 4° ESO (3 students). With the aim of finding out the ambiguity, understandability, difficulty and interpretation of the items, each student was given a scale and was interviewed to evaluate what they thought when they read and answered each item. Later, the questionnaire was revised with the arguments of the interviewed students.

**4) Construct validity**. To obtain the construct validity, an exploratory factor analysis (EFA) was done at first to know the scale’s composition and structure, and secondly a confirmatory factor analysis (CFA) was carried out to verify if the data fit the model extracted from the EFA [68].

**5) Reliability**. The evaluation of the reliability was done via the internal consistency and the time stability of the scale with the test-retest procedure. The internal consistency was calculated by the Cronbach alpha coefficient for each dimension and the total scale, along with the composite reliability (CR). The time stability of the scale, via the test-retest procedure, was performed managing the scale twice with a time interval of two weeks to a subgroup of 50 students. The test-retest reliability was evaluated via the intraclass correlation coefficient (ICC).

**6) External validity**. The convergent validity was studied with the average variance extracted (AVE) and the composite reliability. The discriminant validity was analyzed with the AVE estimated for each construct with the squared interconstruct correlations associated with that factor.

## Recruiting and sample

The sample was obtained via a multi-stage sampling procedure stratified by conglomerates. The sample was based on three criteria, considering as the sample unit the centers where the entrepreneurship education programs were implemented and had had a minimum duration of two academic years.

The education strategies for entrepreneurship applied in the autonomous communities of Spain constitute the first criterion. According to the Oslo Agenda on Education for Entrepreneurship in Europe [69], are three strategies: specific (this covers relevant and necessary aspects for the implementation of entrepreneurship from a predominantly formative perspective, reflecting political priorities); general (this aims to guarantee the coordination between the educational and the business actions, underlining the importance of education as a driver of innovation and entrepreneurship); and isolated initiatives (this seeks to foster certain key elements of the formation of entrepreneurial spirit). Using this criterion, two autonomous communities were selected for each strategy: Andalusia and Galicia, with a specific strategy; Asturias and the Murcia Region, with a general strategy; and Madrid and Catalonia, with isolated initiatives [70].

**Fig 3:**
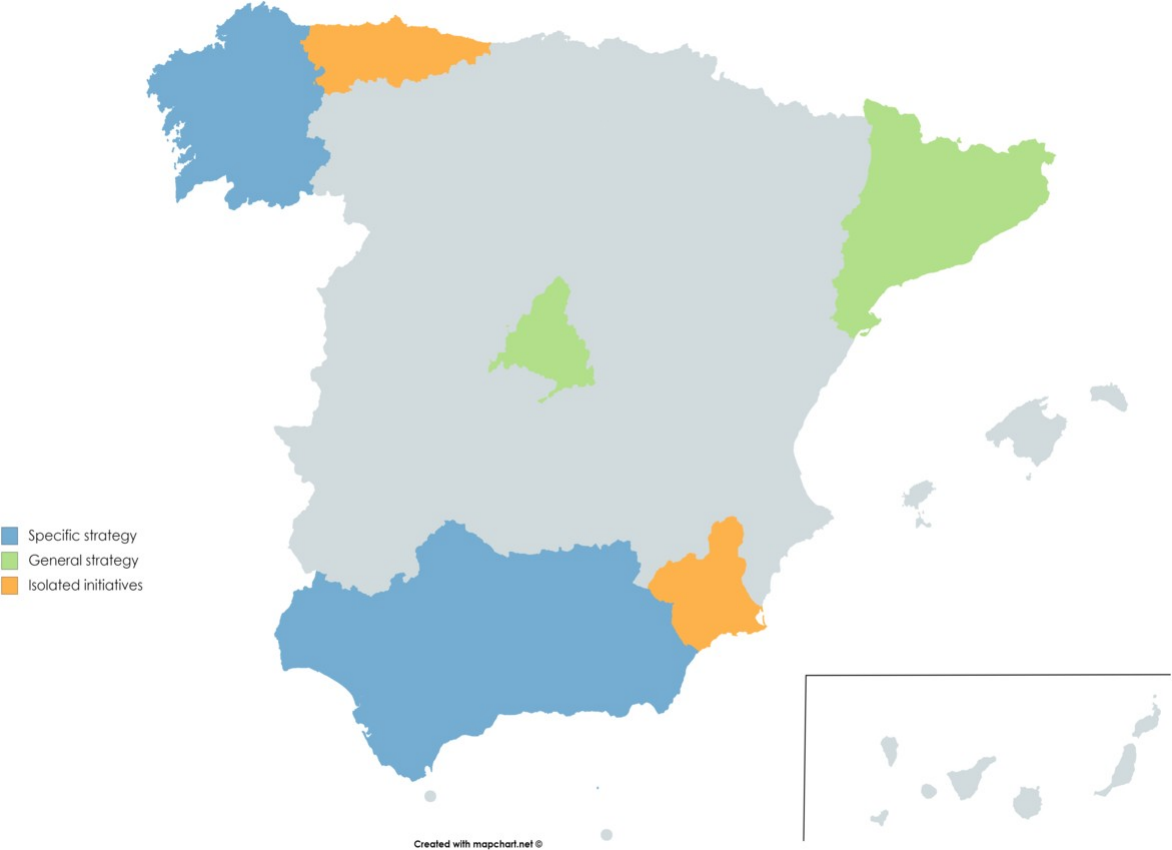
Education strategies for entrepreneurship applied in the autonomous communities. Reprinted from http://mapchart.net under a CC BY license, with permission from Mina Giannekas owner and developer, original copyright 2020.

The second criterion consisted in limiting the study to the education centers supported by public funds given by YEE programs (Young European Enterprise) in ESO, an educational stage which comprises four academic years with an age range between 11 and 16 years old (in the case of the student being a repeater, this can be up to 18 years old). To do so, information was compiled from the databases of the UECOE (Spanish Union of Teaching Cooperatives) and VALNALÓN (a public firm under the authority of the Ministry of Employment, Industry and Tourism of the Government of Asturias), being the databases with more information concerning school centers which implement entrepreneurial education in Spain. 16 educational centers were selected from the six autonomous communities mentioned (Fig 3).

Thirdly, it was established as an inclusion criterion in the research that the educational centers selected had to have a minimum of two academic years in applying the YEE program before our study was done. Under this inclusion criterion, 11 educational centers were selected from the six autonomous communities. No center rejected taking part in the study, the 11 centers chosen making up the total set of school centers which implemented the YEE program. The recruitment of the sample was done from 5th. Abril to 27th. June 2018. The initial sample was composed of the 1473 participants who were at their classes the day of the data collection. However, 33 students were eliminated because they did not fill out the items of the Basic Business Knowledge Scale (BBKS), setting the final sample in 1440 students. The study aim population is 1.964.787 students, corresponding to the total number of students enrolled in ESO in the 2017/2018 academic year in Spain (Ministry of Education, Culture and Sport, split into two in June 2018: Ministry of Education and Vocational training, and Ministry of Culture and Sport). This sample (n = 1440) is statistically representative of the population, assuming an error of 3.4% and a confidence level of 99%. The socio-demographic characteristics of the sample are described in Table 1. The final sample of the study was made up of 679 girls (47.2%) and 761 boys (52.8%), with an age range of 10-17 years old (M = 14.6, SD = 1.597).

**Table 1:** Socio-demographic characteristics of the sample.

			Andalusia	Asturias	Catalonia	Madrid	Murcia Region	Galicia
Age; M			14.02	16.03	16.57	14.86	12.2	13.92
Participants; n (%)			962 (66.80%)	213 (14.80%)	60 (4.16%)	104 (7.22%)	53 (3.69%)	48 (3.33%)
Sex; n (%)	Female		519 (53.96%)	86 (40.37%)	22 (36.67%)	83 (79.81%)	31 (58.50%)	30 (62.5%)
	Male		443 (46.04%)	127 (59.63%)	38 (63.33%)	21 (20.19%)	22 (41.50%)	18 (37.5%)
Education level in ESO; n (%)	1° ESO						38 (71.69%)	
	2° ESO		392 (40.75%)					13 27.08%
	3° ESO		435 (45.22%)	78 (36.61%)		93 (89.42%)		35 (72.92%)
	4° ESO		135 (14.03%)	135 (63.39%)	60 (100%)	11 (10.58%)	15 (28.31%)	
Sex by education level in ESO; n (%)	1° ESO	F					18 (33.97%)	
		M					20 (37.73%)	
	2° ESO	F	240 (24.95%)					9 (18.75%)
		M	152 (15.80%)					4 (8.33%)
	3° ESO	F	213 (22.14%)	26 (12.20%)		38 (36.53%)		20 (41.67%)
		M	222 (23.07%)	52 (24.42%)		55 (52.89%)		15 (31.25%)
	4° ESO	F	66 (6.87%)	60 (28.17%)	48 (80%)		13 (24.52%)	
		M	69 (7.17%)	75 (35.21%)	12 (20%)	11 (10.58)	2 (3.78%)	
Centers with YEE			6	2	2	1	2	2
Centers studied with YEE, n (%)			4 (66.66%)	1 (50%)	2 (100%)	1 (100%)	1 (50%)	2 (100%)
Type of educational center	Public		NA	NA	NA	NA	NA	NA
	Private		NA	NA	NA	NA	NA	NA
	Supported with public funds		4 (100%)	1 (100%)	2 (100%)	1 (100%)	1 (100%)	1 (100%)
Educational strategies for entrepreneurship			Specific	General	Isolated initiatives	Isolated initiatives	General	Specific

^a^ ESO (Educación Secundaria Obligatoria-Compulsory Secondary Education).

^b^ EJE (Empresa Joven Europea-Young European Enterprise).

^c^ In Spain educational centres and students participate voluntarily in entrepreneurial education programs.

## Procedure

The study was done by means of the following procedure: 1) the eligible educational centers were informed about the study and, later, the directors and people in charge of the YEE of the participating centers signed their consent; 2) the management of the educational centers informed the parents or legal tutors about the study and they gave their written consent for the participation of the students under 16 years old; 3) the researchers went to the centers to explain the instructions to fulfill the questionnaire and to help the participants when necessary in the presence of the corresponding tutor; 4) the students were informed of the aim of the study, of their voluntary participation respecting their anonymity and of the confidential treatment of the information provided, thus obtained their written informed consent before completing the test; 5) the students completed the questionnaire online [71] through the Moodle educational platform, within the computer rooms of their respective centers, during a 50-minute session. For this, each participant was given an access code previously coded by the research team. The following is an example of the access code and its corresponding encryption: 1-ALUM-1-ESO-1. Code N° 1 refers to the autonomous community; the ALUM and N° code refers to the number of students surveyed by each autonomous community and the ESO and N° code identifies the educational stage and course of the student.

## Ethical declaration

The Spanish Committee responsible for Ministry of Economy and Competitiveness (MINECO, Government of Spain) reviewed the application, including ethical aspects, and approved the investigation under Grant EDU2013-42936-P. This research was implemented strictly following the current regulations in Spain and Europe in relation with the protection, treatment and free circulation of personal data and regarding the access to scientific information and its preservation [72] [73] [74]. This research was part of student´s standard curricular activities within school hours. The parents or legal tutors gave their written consent for the participation of the students under 16 years old.

## Data analysis

A metric analysis of the 20 items was performed with measures of central tendency (Means), dispersion (Standard Deviation), distribution (Skewness and Kurtosis) and corrected total-item correlations between the item and the dimension to which it belongs. In order to prove the factor validity, the sample was divided into two random subsamples (*N*_1_ + *N*_2_ = 1440). An exploratory factor analysis (EFA) [75] was applied to the first half of the sample (*N*_1_ = 720). The EFA was performed with the Parallel Analysis method (PA), via OLS (Ordinary Least Squares) estimation methods through the ULS (Unweighted Least Squares) technique with direct oblimin rotation (appropriate when correlation between the dimensions to be analyzed is assumed) [76]. The suitability of the sample was studied via the Kaiser-Meyer-Olkin (KMO) and Bartlett’s Sphericity Test [77] [78]. Eigenvalues over 1 and the percentage of variance explained were used to determine the number of factors [79]. Factor loadings above .40 were taken into account as adequate to maintain or eliminate the items. Later, a confirmatory factor analysis (CFA) was done to the second half of the sample (*N*_2_ = 720) to validate the structure of the scale obtained from the EFA [68], using the parallel analysis method with the ULS technique and direct oblimin rotation. Following the recommendations of Hair, Black, Babin and Anderson [80] and that of Hoyle [81], and due to the value of *χ*^2^ being very sensitive to small variations of the hypothesized model when one is working with large samples [82], we used a combined strategy of different goodness-of-fit indices: a) good fit values if the Goodness of Fit Index GFI, Comparative Fit Index CFI ≥ .96, the Tucker-Lewis Index TLI ≥ .95, the Root Mean Squared Error of Approximation RMSEA ≤ .05; b) moderate fit values if the CFI, GFI and TLI ≥ .90, RMSEA ≤ .08; c) poor values if the CFI, GFI and TLI ≥ .90, RMSEA ≤ .10.); and d) the Standardized Root Mean Square Residual SRMSR with values of .08 or lower indicate a good fit [80] [82].

The reliability analysis was done for each dimension via the Cronbach alpha, the Composite Reliability (CR) with values over .7 as an indicator of good internal consistency [83] and the intraclass correlation coefficient where values over .75 were considered acceptable [84] [85]. The evidence of the external validity was based on the convergent and discriminant validity. The convergent validity has been validated via the average variance extracted (AVE). As a criterion of acceptance, the AVE has to be .50 or greater [86] and the CR equal to .7. The discriminant validity between constructs was established via the square root of the AVE, which must be greater than the correlation between the constructs [86]. The statistical analyses were done with the statistic packets: FACTOR 10.4 [87]; EQS 6.2 [88]; Smart PLS 2.0 M3 [89] and SPSS 23.0 [90].

## Results

### 1) BBKS metric item analysis

At first, the averages range from 1.98 to 2.75. Standard Deviation is above 1 considered good [91]. Skewness has been between -1.28 and 1.12, and Kurtosis scores between -1.44 and 0.23. Considering for both statistics that the values within the range between -1.5 and +1.5 are adequate [92] [93] [94]. Secondly, the corrected total item correlations between the item and the dimension to which it is assumed to belong are above .45, being considered adequate values [95] (Table 2).

**Table 2:** Item descriptive statistics.

Items No.	Descriptions of the items	M	SD	Skew.	Kurt.	I-tcd.
1	The meaning of “business environment”.	2.43	1.57	0.70	-0.48	0.63
2	The concept of “business opportunities”.	2.28	1.17	1.12	0.23	0.45
3	Benchmarking techniques and SWOT.	2.07	1.09	0.52	-1.09	0.65
4	The meaning of the term “customer selection”.	2.55	1.09	-1.28	-1.29	0.55
5	The characteristics of a potential customer.	2.34	1.12	0.15	-1.37	0.59
6	Advantages and disadvantages of products/services existing in the market.	2.22	1.16	0.35	-1.36	0.59
7	Types of firm (cooperative, limited company, workforce owned company, etc.).	2.11	1.09	0.44	-1.16	0.64
8	The minimum capital necessary to set up a firm.	2.04	1.11	0.57	-1.11	0.61
9	The business responsibilities of the partners in a firm.	2.16	1.10	0.36	-1.26	0.65
10	The process and procedures for setting up a firm.	2.43	1.12	0.51	-1.38	0.60
11	The organizational structure of a firm (areas, managerial posts, etc.).	2.75	1.06	-0.36	-1.10	0.66
14	The elements of an economic-financial plan.	2.60	1.16	-0.17	-1.44	0.63
15	What the accounting of a firm consists of.	1.98	1.09	0.63	-1.03	0.57
16	The financial processes of a firm.	2.25	1.14	0.25	-1.38	0.57
17	The technical characteristics of a firm’s products/services.	2.08	1.05	0.47	-1.06	0.65
19	What is a logotype, how is it made and what is it for?	2.41	1.12	0.06	-1.37	0.72
22	The meaning and principles of the expression “business social responsibility”.	2.27	1.13	0.23	-1.36	0.75
23	What “ethical code” means.	2.32	1.13	0.18	-1.39	0.73
24	The meaning of “stakeholders”	2.58	1.15	-0.12	-1.42	0.67
25	The components of a plan of Business Social Responsibility.	2.30	1.08	0.18	-1.28	0.72

Mean = M. Standard Deviation = SD. Skewness = Skew. Kurtosis = Kurt. Item-total correlations dimension = I-tcd.

### 2) Construct validity

#### Exploratory factor analysis (EFA)

An EFA was performed with the first half of the sample (*N*_1_ = 720), where the Kaiser-Meyer-Olkin (KMO = 0.93) and the Bartlett´s Test of Sphericity (*χ*^2^ = 8608,1; p < 0.001) showed their suitability for the factor analysis. The scale of basic business knowledge is made up of three factors with 20 items. Two items with loadings below .40 were eliminated (KBM 17 and KBM 19). The exploratory solution derived from the EFA is, as we have described before, composed of three factors:

1. Knowledge in Business Management (KBM): they are those relating to the development of productivity, competitiveness and viability of the company in the most efficient and effective possible way.
2. Legal Knowledge (LK): they are those relating to the legal and administrative aspects associated with the constitution of a company.
3. Strategic Knowledge (SK): these refer to the business context to establish a competitive advantage or difference in the products and/or services offered by the company in relation to its competitors.

The EFA was constructed with a total of 18 items in three dimensions (Table 3).

**Table 3:** Dimensions of basic business knowledge.

Dimensions	N° of Items
Knowledge in Business Management (KBM)	9 items and 2 eliminated = 7 items
Legal Knowledge (LK)	5 items
Strategic Knowledge (SK)	6 items
Total: 3 dimensions	18 items

The goodness-of-fit indicators indicated the suitability of the results: GFI = .99, CFI = .98, TLI = .97, RMSEA = .05 and SRMSR = .03. The percentage of total variance explained was 60.3%. The first factor, KBM, obtained 36.9% and is comprised of 7 items. The second factor, LK, explains 15.1% and includes 5 items. The third factor, SK, explains 8.3% and is made up of 6 items. The factor loadings ranged from .869 to .416 and the eigenvalues of greater than 1. The results of the EFA with the factor loadings, eigenvalues and explained variance are shown in Table 4.

**Table 4:** Factor loadings results and communalities h^2^ retained after exploratory factor analysis (n = 720).

Items	Description of the factors and items	Factors Loading			h^2^
**F1. Knowledge in Business Management (KBM)**		**1**	**2**	**3**	
KBM 24	The meaning of “stakeholders”.	.869			.766
KBM 3	Benchmarking techniques and SWOT.	.851			.785
KBM 25	The components of a plan of Business Social Responsibility.	.646			.500
KBM 23	What “ethical code” means.	.582			.534
KBM 16	The financial processes of a firm.	.497			.725
KBM 15	What the accounting of a firm consists of.	.485			.650
KBM 14	The elements of an economic-financial plan.	.485			.576
KBM 17	The technical characteristics of a firm’s products/services.	.319			.750
KBM 19	What is a logotype, how is it made and what is it for?	.310			.677
**F2. Legal Knowledge (LK)**					
LK 8	The minimum capital necessary to set up a firm.		.715		.455
LK 9	The business responsibilities of the partners in a firm.		.610		.456
LK 11	The organizational structure of a firm (areas, managerial posts, etc.).		.599		.454
LK 7	Types of firm (cooperative, limited company, workforce owned company, etc.).		.561		.470
LK 10	The process and procedures for setting up a firm.		.544		.517
**F3. Strategic Knowledge (SK)**					
SK 2	The concept of “business opportunities”.			.666	.457
SK 1	The meaning of “business environment”.			.626	.438
SK 4	The meaning of the term “customer selection”.			.594	.468
SK 5	The characteristics of a potential customer.			.459	.486
SK 6	Advantages and disadvantages of products/services existing in the market.			.424	.401
SK 22	The meaning and principles of the expression “business social responsibility”.			.416	.511
**Eigenvalue**		18.35	7.45	4.5	-
**Explained Variance (%)**		36.9	15.1	8.3	-
**Cumulative Variance (%)**		36.9	52	60.3	-

h^2^ Communalities.

#### Confirmatory factor analysis (CFA)

**Fig 4:**
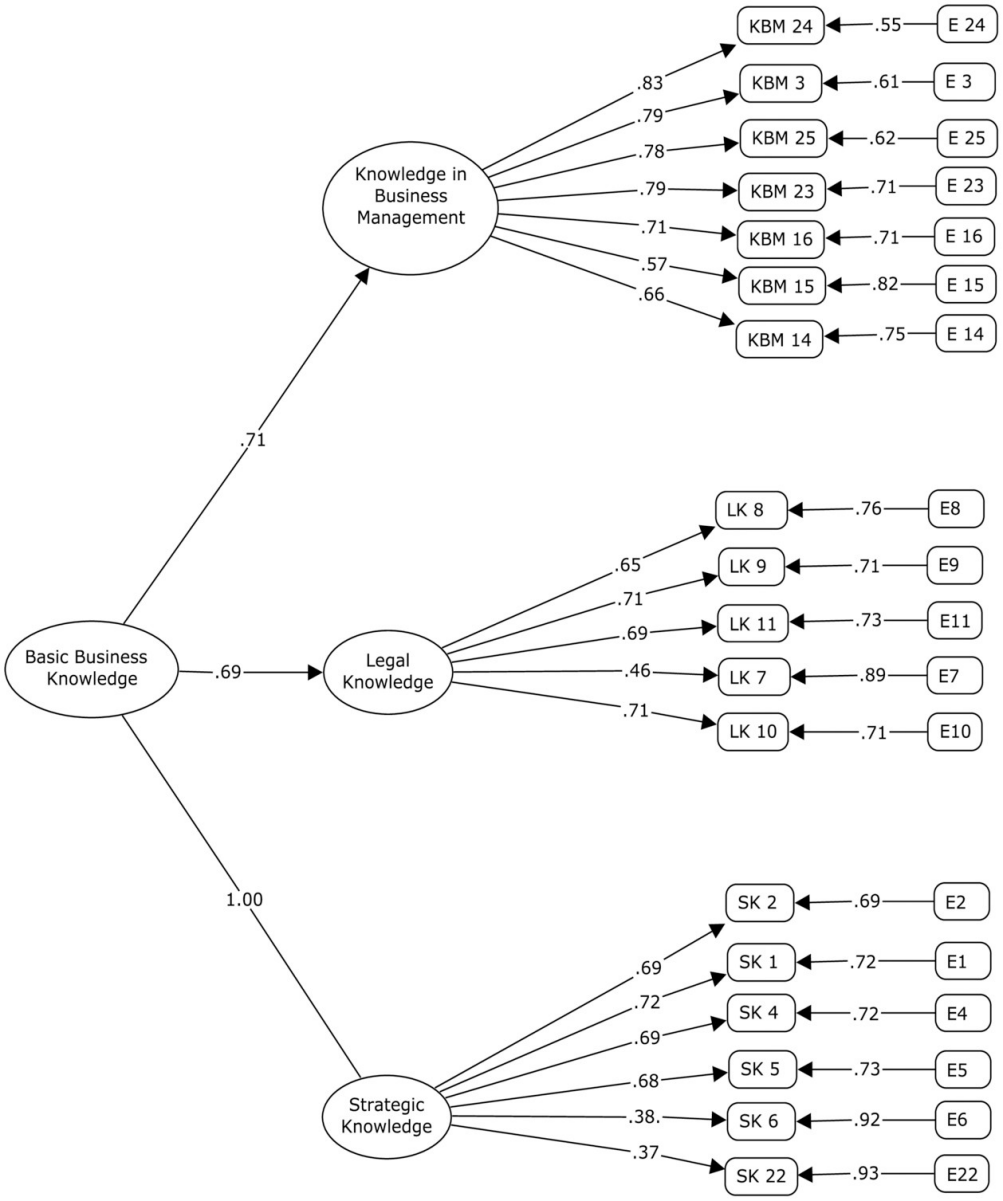
Second-order confirmatory factor analysis factor loadings construct validity for the Basic Business Knowledge Scale (BBKS). n = 720; All factor loadings are standardized and are statistically significant, p < 0.001.

The CFA was done with the second half of the sample (*N*_2_ = 720). It was applied via a second-order factor analysis where the items were analyzed and it was determined if the three dimensions configured the Basic Business Knowledge Scale (BBKS). All the factor loadings were statistically significant (p < 0.001). The fit indices GFI = .99, CFI = .99, TLI = .99, RMSEA = .03 and SRMSR = .02 showed the good fit of the proposed model [96] (Fig 4).

### 3) Reliability

The Cronbach alpha coefficient for each of the domains varied between .76 and .83. The AVE scores were above .50 and the composite reliability has been greater than the criterion of .70. The intraclass correlation coefficient was .92. The results obtained in all the dimensions, which exceed the minimum recommended values [97] [80], indicate that the scale has a satisfactory stability (Table 5).

**Table 5:** Reliability and validity for the Basic Business Knowledge Scale and its dimensions.

Dimensions	Items	CR	AVE	Cronbach´s n = 720	ICC n = 50
Knowledge in Business Management	24, 3, 25, 23, 16, 15, 14	.88	.59	.83	.83
Legal Knowledge	8, 9, 11, 7, 10	.85	.53	.78	.81
Strategic Knowledge	2, 1, 4, 5, 6, 22	.83	.56	.76	.86
Basic Business Knowledge Scale (total)	18	-	-	.80	.92

Construct Reliability = CR. Average Variance Extracted = AVE. Cronbach´s = Cronbach´s Alpha. Intraclass Correlation Coefficient = ICC.

### 4) External validity

As to the convergent validity, all the factors present values above .7 in the CR and the AVE is greater than .50. This indicates the appropriateness of the items in the measuring of the dimensions [98] (Table 5). As to the discriminant validity, the values of the square root of the AVE are greater than the correlations with the other constructs. The independence of the latent variables is thus confirmed [99] (Table 6).

**Table 6:** Discriminant validity.

Dimensions	Factor 1	Factor 2	Factor 3
Knowledge in Business Management	.702		
Legal Knowledge	.517	.730	
Strategic Knowledge	.645	.577	.684

Square root of the AVE in italics in the diagonal.

## Discussion

To educate in and for entrepreneurship has different dimensions (economic, cultural, social, ethical, political, etc.) which makes this field a complex and difficult area to outline [100]. This complexity partially explains the difficulty of attaining a consensus concerning a specific educational model. Hence, different proposals with indictors and evaluative mechanisms arise [101]. Along with a strictly cognitive dimension, business competence possibly requires the inclusion of meta-cognitive, affective and motivational aspects [102] [103]. This is similar to the research activities developed in the theoretical analysis of the “learn to learn” construct [104] [105], a requisite of another of the key competences pointed out by the European Union since 2007. Another dimension, linked to the social and relational area, could even be aggregated [106] [107].

While research pursues its advances seeking the best formative and evaluative model of business competence possible [108] [21], as a complex construct, the partial contributions in this direction are shown relevant for the theory and the practice of entrepreneurship education, even promoting the future construction of more holistic and complex instruments.

This research has developed the study of the validity and reliability of a scale to measure the basic business knowledge of students in the stage of Compulsory Secondary Education. The curricular integration of entrepreneurship education in the compulsory stages is not being as effective as would be desirable for the development of business spirit. In the GEM 2018-2019 Report [109], in relation with the conditions for entrepreneurship at a global level, the experts value business education and formation in the compulsory education stage as being very low. Regarding this, the evaluation of business competence in the Compulsory Secondary Education stage is an area that has hardly been developed and which lacks valid and reliable instruments [70]. Although instruments which measure the intention, attitude or entrepreneurial personality risks in adolescents have been recently designed [110] [111] [112] [113] [114], there are not assessment instruments that measure the business knowledge which students should acquire in this educational stage.

There were 20 items left to study the construct validity after the initial phases of the identification of the dimensions, the generation of items, and content and face validity. In the exploratory factor analysis (EFA), 18 items were extracted with satisfactory factor loadings and 2 were not kept because they had low factor loadings: KBM 17 “The technical characteristics of a firm’s products/services” and KBM 19 “What is a logotype, how is it made and what is it for?”. The exploratory factor solution obtained from the 18 items was the extraction of three factors: Knowledge in Business Management (KBM), Legal Knowledge (LK) and Strategic Knowledge (SK). The factor loadings of the items related with each of the factors were satisfactory and the fit indices were suitable.

Later, a confirmatory factor analysis was done where a single factor called “Basic Business Knowledge” was extracted, obtaining a more parsimonious factor solution. The cut-off points of the different fit indices –the CFI, GFI, TLI, RMSEA and SRMSR- showed acceptable values, confirming a unifactorial structure of business knowledge for adolescents configured by three dimensions identified in the EFA: KBM with 7 items, LK with 5 items and SK with 6 items. The reliability of the three factors and of the whole scale has been above .75, being pertinent for its use in research [115]. The indicators of composite reliability of each factor also revealed very appropriate values, while average variance explained was over .55, higher than the .50 recommended by Hair [116]. Besides, the external validity fulfilled the established criteria. Hence, the results confirm a model with a structure made up of three first-order factors (KBM, LK and SK) and a second-order factor called Basic Business Knowledge (BBK).

Research evinces a more complex configuration about the composition of business knowledge, especially in the post-compulsory stages. However, this study delimits the number of dimensions of business knowledge in the stage of Compulsory Secondary Education into three areas: management, jurisprudence and business strategy. Although it could seem to be an infra-representation of business knowledge, it is rather an adaptation to the business contents corresponding to this educational stage, in accordance with the “Progression Model” of business education in the European Union [117]. This explains the absence of other necessary dimensions of business knowledge more suited to higher education contents in the structure of our Scale, but perhaps inappropriate for the conformation of a basic structure, primarily constituting the indispensable cultural foundations of entrepreneurship [118]. Compulsory Secondary Education is a key stage for acquiring business knowledge as it means the link between the initial business education of primary education and the more specific education of post-compulsory studies. An appropriate evaluation concerning the students’ knowledge will beneficially redound in the acquiring of the entrepreneurial culture needed for the formation of a business identity. Due to all this, the development of instruments which facilitate the metric of the basic knowledge of entrepreneurship is required, improving its diagnosis and permanent valuation.

In this respect, the results suggest that the Basic Business Knowledge Scale is a valid and reliable instrument that has enabled the identification of the dimensions of business knowledge in adolescents in the Compulsory Secondary Education stage (S5 File). This instrument contributes to the reduction of the evaluative gap which hinders a better understanding of the configuration of the business identity in ages with a high level of cultural permeability. In this sense, the Scale could be used along with other instruments linked to the business domain to understand how business identity is formed or to diagnose and detect the students’ formative needs.

Although the study has contributed verified evidence about its validity and reliability, this research has certain limitations: a) the sex variable has not been taken into account when carrying out the study; b) social and demographic variables which may influence the formation of business knowledge have not been studied; c) the study has been centered on a specific age range, type of school and business education program. It would thus be advisable for future research to be carried out with students of a different age group and sex, in different schools and business education programs, as well as in other social and economic contexts. Furthermore, our Scale could be expanded in future researches by including elements of the economic logic of human action.

## Conclusion

The findings of this research offer evidence concerning the validity and reliability of the Basic Business Knowledge Scale in students of the Compulsory Secondary Education stage. This Scale, as well as being useful for metric contributions in this area, is an appropriate tool for the diagnosis and the evaluation of basic business knowledge in entrepreneurship programs, particularly of business education in the secondary education stage.

## Supporting information

S1 File PRISMA checklist. (PDF)

S2 File Interview guides in English version. (PDF)

S3 File Interview guides in Spanish version. (PDF)

S4 File Basic Business Knowledge Scale for Secondary Education Students (BBKS)- Spanish version. (DOC)

S5 File Basic Business Knowledge Scale for Secondary Education Students (BBKS)- English version. (DOCX)
