# Supplementary material for: Basic business knowledge scale for secondary education students. Development and validation with Spanish teenagers
Source: PLoS One. 2020 Jul 7;15(7):e0235681. doi: 10.1371/journal.pone.0235681 (PMC7340510; doi:10.1371/journal.pone.0235681)
Supplement: S2 File — (PDF) [file pone.0235681.s002.pdf]

INTERVIEW FOR EXPERTS IN ENTREPRENEURSHIP EDUCATION IN THE STAGES OF COMPULSORY EDUCATION

Interviewer's Topic Guide

Introduction to Interview

First of all, we express our appreciation for your participation.

With this interview we try to know your opinion, as an expert teacher in entrepreneurship education, about knowledge concerning the programs of this type of education. Your valuable information will help us to design an assessment instrument for students´ basic business knowledge, which is part of an extensive research on entrepreneurship education in the stages of compulsory education.

Comment about confidentiality:

This interview will be recorded and later transcribed, guaranteeing that all the data from the conversation will be confidential and anonymous. With your permission, we will begin recording.

Start of the interview

Conceptualization of business knowledge of entrepreneurship education programs

1) Could you define what business knowledge is?

2) Could you describe which business knowledge you teach in entrepreneurship education programs?

3) Could you tell me the characteristics of this business knowledge?

Assessment of business knowledge of entrepreneurship education programs

From your perspective as a teacher:

4) Which are the most relevant business knowledge to teach? Why?

5) Which are the least relevant business knowledge to teach? Why?

6) Do you think the business knowledge you teach is useful for the professional development of students? Why?

7) Apart from this knowledge, could other types of business knowledge be imparted?

7.1) If yes, why? and which ones?

7.2) If not, why can't another type of business knowledge be imparted?

Teaching experience and business knowledge of entrepreneurship education programs

From your experience as a teacher:

8) Would it be convenient to teach students all the business knowledge about the design of a project/business plan?

If not, which ones would you teach? Why?

If yes, why?

9) If you did not have time to teach all the business knowledge of the program, what content would you prioritize to teach your students? Why?

10) Thinking of compulsory secondary education as an educational stage of basic business training, what business knowledge would you teach students for their subsequent incorporation into vocational training or pre-university studies?

11) What are the criteria you use to select and prioritize business knowlege for students?

12) Thinking about your teaching experience and the business context, do you think that the program's business knowledge are adapted to the training needs of companies? Why?

Closure of the interview

We have no further questions, although we would like to offer you the opportunity to add any comments or observations about the business knowledge that are being taught, if you wish.

We reiterate our gratitude for your participation.

INTERVIEW FOR SECONDARY EDUCATION STUDENTS PARTICIPATING IN ENTREPRENEURSHIP EDUCATION PROGRAMS

Interviewer's Topic Guide

Introduction to Interview

First of all, we express our appreciation for your participation.

With this interview we try to know your opinion, as an expert teacher in entrepreneurship education, about knowledge concerning the programs of this type of education. Your valuable information will help us to design an assessment instrument for students´ basic business knowledge, which is part of an extensive research on entrepreneurship education in the stages of compulsory education.

Comment about confidentiality:

This interview will be recorded and later transcribed, guaranteeing that all the data from the conversation will be confidential and anonymous. There are no right or wrong answers. You are completely free to express your opinion and you can request any clarification you need during the interview. With your permission, we will begin recording.

Start of the interview

Typology of business knowledge in entrepreneurship education programs

As a student:

1) What business knowledge are learned in the entrepreneurship education program?

2) Which would be the business knowledge needed to develop a business project or plan?

From your perspective:

3) Which are the most important business knowledge you've learned? Why?

4) Which are the least important business knowledge you've learned? Why?

You've participated in entrepreneurship education programs in previous courses, thinking about them:

5) What type of business knowledge did you learn in those programs?

5.1) Are they linked to the knowledge you are learning in this academic year?

5.2) In which way?

5.3) Could you give any examples of the link or relationship between business knowledge from previous years and the current ones?

5.4) Considering everything you have learned, how would you classify the acquired knowledge?

Characteristics of business knowledge in entrepreneurship education programs

From your perspective as a student:

6) What is business knowledge? How would you define it?

7) In general, how are the business knowledge you know?

More concretely:

7.1) Are they linked to real life? Can you give any examples or describe the degree of concreteness or abstraction of this knowledge?

7.2) Do you think that the business knowledge learned are immediately applicable to the company? Why? Can you give any examples or describe which knowledge you think is more applicable and which is not?

7.3) Do you want to highlight any more characteristics of this business knowledge?

Comparing the business knowledge of the entrepreneurship education program of this academic year with the entrepreneurship education programs of previous courses:

8) Which are the differences or similarities of this business knowledge?

8.1) Could you describe some examples of the differences or similarities?

9) Could you describe the phases/stages of a business project or plan?

9.1) Could you give examples of those phases/stages?

9.2) With the knowledge gained, would you dare to start a business? Would any unacquired knowledge be required?

Closure of the interview

We have no further questions, although we would like to offer you the opportunity to add any comments or observations about the business knowledge that are being taught, if you wish.

We reiterate our gratitude for your participation.
