# Supplementary material for: Basic business knowledge scale for secondary education students. Development and validation with Spanish teenagers
Source: PLoS One. 2020 Jul 7;15(7):e0235681. doi: 10.1371/journal.pone.0235681 (PMC7340510; doi:10.1371/journal.pone.0235681)
Supplement: S3 File — (PDF) [file pone.0235681.s003.pdf]

ENTREVISTA PARA EXPERTOS EN EDUCACIÓN EMPRESARIAL EN LAS ETAPAS DE LA EDUCACIÓN OBLIGATORIA

Guía temática del entrevistador

Introducción a la entrevista

En primer lugar, le expresamos nuestro agradecimiento por su participación.

Con esta entrevista pretendemos conocer su opinión, como docente experto en educación empresarial, sobre los conocimientos que forman parte de los programas de este tipo de educación. Su valiosa información nos será útil para diseñar un instrumento de evaluación sobre conocimientos empresariales básicos del alumnado, que forma parte de una amplia investigación sobre la educación empresarial en las etapas de la educación obligatoria.

Comentario sobre la confidencialidad:

Esta entrevista será grabada y posteriormente transcrita, garantizándole que todos los datos de la conversación serán confidenciales y anónimos. Con su permiso, vamos a comenzar a grabar.

Inicio de la entrevista

Conceptualización de los conocimientos empresariales de los programas de educación emprendedora

1) ¿Podría definirme qué son los conocimientos empresariales?

2) ¿Me puede describir cuáles son los conocimientos empresariales que enseña en los programas de educación emprendedora?

3) ¿Me podría decir las características de esos conocimientos empresariales?

Valoración de los conocimientos empresariales de los programas de educación emprendedora

Desde su punto de vista como docente:

4) ¿Cuáles son los conocimientos empresariales más relevantes para enseñar? ¿Por qué?

5) ¿Cuáles son los conocimientos empresariales menos relevantes para enseñar? ¿Por qué?

6) ¿Cree que los conocimientos empresariales que enseña son útiles para el desarrollo profesional del alumnado? ¿Por qué?

7) ¿Aparte de estos conocimientos, se podría impartir otro tipo de conocimientos empresariales?

7.1) En caso afirmativo, ¿por qué? y ¿cuáles?

7.2) En caso negativo, ¿por qué no se puede impartir otro tipo de conocimientos empresariales?

Experiencia docente y conocimientos empresariales de los programas de educación emprendedora

Desde su experiencia como docente:

8) ¿Sería conveniente enseñar al alumnado todos los conocimientos empresariales sobre el diseño de un proyecto/plan de empresa?

En caso negativo, ¿cuáles enseñaría? ¿Por qué?

En caso afirmativo, ¿por qué?

9) En el caso de que no tuviera tiempo para enseñar todos los conocimientos empresariales del programa, ¿qué contenidos priorizaría para enseñar a sus alumnos? ¿Por qué?

10) Pensando en la Educación Secundaria Obligatoria como etapa educativa de formación empresarial básica, ¿qué conocimientos empresariales enseñaría al alumnado para su posterior incorporación a la Formación Profesional o al Bachillerato?

11) ¿Cuáles son los criterios que utiliza para seleccionar y priorizar los conocimientos empresariales destinados al alumnado?

12) Pensando en su experiencia docente y en el contexto empresarial, ¿piensa que los conocimientos empresariales del programa se adecuan a las necesidades formativas de las empresas? ¿Por qué?

Cierre de la entrevista

No tenemos más preguntas, aunque nos gustaría ofrecerle la oportunidad de añadir algún comentario u observación sobre los conocimientos empresariales que son objeto de enseñanza, si así lo desea.

Le reiteramos nuestro agradecimiento por su participación.

ENTREVISTA PARA ESTUDIANTES DE EDUCACIÓN SECUNDARIA PARTICIPANTES EN PROGRAMAS DE EDUCACIÓN EMPRESARIAL

Guía temática del entrevistador

Introducción a la entrevista

En primer lugar, le expresamos nuestro agradecimiento por su participación.

Con esta entrevista pretendemos conocer su opinión, como alumno participante en programas de educación empresarial, sobre los conocimientos que forman parte de los programas de este tipo de educación. Su valiosa información nos será útil para diseñar un instrumento de evaluación sobre conocimientos empresariales básicos del alumnado, que forma parte de una amplia investigación sobre la educación empresarial en las etapas de la educación obligatoria.

Comentario sobre la confidencialidad:

Esta entrevista será grabada y posteriormente transcrita, garantizándole que todos los datos de la conversación serán confidenciales y anónimos. No existen respuestas correctas o incorrectas. Tiene total libertad para expresar su opinión y durante la entrevista puede solicitar la aclaración que precise. Con su permiso, vamos a comenzar a grabar.

Inicio de la entrevista

Tipología de los conocimientos empresariales en los programas de educación emprendedora

Como estudiante:

1) ¿Qué conocimientos empresariales se aprenden en el programa de educación emprendedora?

2) ¿Cuáles serian los conocimientos empresariales necesarios para desarrollar un proyecto/plan de empresa?

Desde su punto de vista:

3) ¿Cuáles son los conocimientos empresariales más importantes que ha aprendido? ¿Por qué?

4) ¿Cuáles son los conocimientos empresariales menos importantes que ha aprendido? ¿Por qué?

Ha participado en programas de educación emprendedora en cursos anteriores, pensando en ellos:

5) ¿Qué clase de conocimientos empresariales aprendió en esos programas?

5.1) ¿Están vinculados a los conocimientos que está aprendiendo en este curso académico?

5.2) ¿De qué modo?

5.3) ¿Podría poner ejemplos de la vinculación o relación de los conocimientos empresariales de años anteriores con los actuales?

5.4) Considerando todo lo aprendido, ¿cómo clasificaría los conocimientos adquiridos?

Características de los conocimientos empresariales en los programas de educación emprendedora

Desde su punto de vista como estudiante:

6) ¿Qué son los conocimientos empresariales? ¿Cómo los definiría?

7) De forma general, ¿Cómo son los conocimientos empresariales que conoce?

Más concretamente:

7.1) ¿Están vinculados a la vida real? ¿Puede poner ejemplos o describir el grado de concreción o de abstracción de dichos conocimientos?

7.2) ¿Cree que los conocimientos empresariales aprendidos son aplicables de forma inmediata a la empresa? ¿Por qué? ¿Puede poner ejemplos o describir qué conocimientos cree que son más aplicables y cuáles no?

7.3) ¿Quiere destacar alguna característica más de esos conocimientos empresariales?

Comparando los conocimientos empresariales del programa de educación empresarial de este curso académico con los programas de educación empresarial de cursos anteriores:

8) ¿Cuáles son las diferencias o similitudes de esos conocimientos empresariales?

8.1) ¿Podría describir algunos ejemplos de las diferencias o similitudes?

9) ¿Podría describir las fases/etapas de un proyecto/plan de empresa?

9.1) ¿Podría poner ejemplos de esas fases /etapas?

9.2) Con los conocimientos adquiridos, ¿se atrevería a iniciar un negocio? ¿Haría falta algún conocimiento no adquirido?

Cierre de la entrevista

No tenemos más preguntas, aunque nos gustaría ofrecerle la oportunidad de añadir algún comentario u observación sobre los conocimientos empresariales que son objeto de enseñanza, si así lo desea.

Gracias por participar en esta entrevista.
