# Supplementary material for: Basic business knowledge scale for secondary education students. Development and validation with Spanish teenagers
Source: PLoS One. 2020 Jul 7;15(7):e0235681. doi: 10.1371/journal.pone.0235681 (PMC7340510; doi:10.1371/journal.pone.0235681)
Supplement: S4 File — (DOC) [file pone.0235681.s004.doc]

**ESCALA BÁSICA DE CONOCIMIENTOS EMPRESARIALES PARA ESTUDIANTES DE EDUCACIÓN SECUNDARIA**

Lea cuidadosamente cada cuestión y marque su respuesta. Por favor, marque una sola respuesta en cada cuestión y responda a todas las cuestiones, según las siguientes respuestas.

2. Se ha trabajado en clase y creo que lo he aprendido. (Trabajado y aprendido, TA).

1. Creo que se ha trabajado en clase, pero no lo he aprendido. (Trabajado, pero no aprendido, TNA).

0. Creo que no se ha trabajado en clase y no lo he aprendido (No trabajado, NT).

|  | ITEMS | TA | TNA | NT |
| --- | --- | --- | --- | --- |
|  |  | 2 | 1 | 0 |
| 1 | El significado de “stakeholders” o “grupos de interés”. |  |  |  |
| 2 | Las técnicas Benchmarking y DAFO. |  |  |  |
| 3 | Los componentes de un plan de Responsabilidad Social Empresarial. |  |  |  |
| 4 | Lo que significa un “código ético”. |  |  |  |
| 5 | Los procesos financieros de una empresa. |  |  |  |
| 6 | En qué consiste la contabilidad de una empresa. |  |  |  |
| 7 | Los elementos de un plan económico-financiero. |  |  |  |
| 8 | El capital mínimo necesario para montar una empresa. |  |  |  |
| 9 | Las responsabilidades empresariales de los socios en una empresa. |  |  |  |
| 10 | La estructura organizativa de una empresa (áreas, cargos directivos…) |  |  |  |
| 11 | Tipos de empresa (cooperativa, sociedad anónima, sociedad laboral…) |  |  |  |
| 12 | El proceso y trámites para la constitución de una empresa. |  |  |  |
| 13 | El concepto de “oportunidades empresariales”. |  |  |  |
| 14 | El significado de “entorno empresarial”. |  |  |  |
| 15 | El significado del término “selección de clientes”. |  |  |  |
| 16 | Las características de un cliente potencial. |  |  |  |
| 17 | Ventajas y desventajas de productos/servicios existentes en el mercado. |  |  |  |
| 18 | El sentido y principios de la expresión “responsabilidad social empresarial”. |  |  |  |

Gracias, por cumplimentar este cuestionario.
