# Supplementary material for: Basic business knowledge scale for secondary education students. Development and validation with Spanish teenagers
Source: PLoS One. 2020 Jul 7;15(7):e0235681. doi: 10.1371/journal.pone.0235681 (PMC7340510; doi:10.1371/journal.pone.0235681)
Supplement: S5 File — (DOCX) [file pone.0235681.s005.docx]

**BASIC BUSINESS KNOWLEDGE SCALE FOR SECONDARY EDUCATION STUDENTS**

Carefully read each question and mark your answer. Please check only one answer in each question and answer all questions, according to the following answers.

2. It has been worked on at class and I think I’ve learnt it. (Worked and learned, WL).

1. I think it has been worked on at class, but I haven’t learnt it. (Worked but not learned, WNL).

0. I think it hasn’t been worked on at class and I haven’t learnt it. (Not worked, NW).

|  | ITEMS | WL | WNL | NW |
| --- | --- | --- | --- | --- |
|  |  | 2 | 1 | 0 |
| 1 | The meaning of “stakeholders”. |  |  |  |
| 2 | Benchmarking techniques and SWOT. |  |  |  |
| 3 | The components of a plan of Business Social Responsibility. |  |  |  |
| 4 | What “ethical code” means. |  |  |  |
| 5 | The financial processes of a firm. |  |  |  |
| 6 | What the accounting of a firm consists of. |  |  |  |
| 7 | The elements of an economic-financial plan. |  |  |  |
| 8 | The minimum capital necessary to set up a firm. |  |  |  |
| 9 | The business responsibilities of the partners in a firm. |  |  |  |
| 10 | The organizational structure of a firm (areas, managerial posts, etc.) |  |  |  |
| 11 | Types of firm (cooperative, limited company, workforce owned company, etc.) |  |  |  |
| 12 | The process and procedures for setting up a firm. |  |  |  |
| 13 | The concept of “business opportunities”. |  |  |  |
| 14 | The meaning of “business environment”. |  |  |  |
| 15 | The meaning of the term “customer selection”. |  |  |  |
| 16 | The characteristics of a potential customer. |  |  |  |
| 17 | Advantages and disadvantages of products/services existing in the market. |  |  |  |
| 18 | The meaning and principles of the expression “business social responsibility”. |  |  |  |

Thank you for completing the questionnaire.
